# Intrinsic Multi-Scale Dynamic Behaviors of Complex Financial Systems

**DOI:** 10.1371/journal.pone.0139420

**Published:** 2015-10-01

**Authors:** Fang-Yan Ouyang, Bo Zheng, Xiong-Fei Jiang

**Affiliations:** 1 Department of Physics, Zhejiang University, Hangzhou 310027, China; 2 School of Electronics and Information, Zhejiang University of Media and Communications, Hangzhou 310018, China; 3 Collaborative Innovation Center of Advanced Microstructures, Nanjing 210093, China; University of Rijeka, CROATIA

## Abstract

The empirical mode decomposition is applied to analyze the intrinsic multi-scale dynamic behaviors of complex financial systems. In this approach, the time series of the price returns of each stock is decomposed into a small number of intrinsic mode functions, which represent the price motion from high frequency to low frequency. These intrinsic mode functions are then grouped into three modes, i.e., the fast mode, medium mode and slow mode. The probability distribution of returns and auto-correlation of volatilities for the fast and medium modes exhibit similar behaviors as those of the full time series, i.e., these characteristics are rather robust in multi time scale. However, the cross-correlation between individual stocks and the return-volatility correlation are time scale dependent. The structure of business sectors is mainly governed by the fast mode when returns are sampled at a couple of days, while by the medium mode when returns are sampled at dozens of days. More importantly, the leverage and anti-leverage effects are dominated by the medium mode.

## Introduction

In recent years, there has been a growing interest of physicists in complex financial systems. Physical concepts and methods have been applied to analyze the dynamic behaviors in financial markets, which are important examples of complex systems with many-body interactions. As large amounts of historical financial data have piled up in stock markets, it allows to explore the fine structure of the financial dynamics and achieve various empirical results [1–10]. Very recently, with the online big data, various new methods are proposed and the results are augmented. For examples, the price change could be predicted by using the collective mood states derived from Twitter [11], and the trading behavior may be quantified with the Google Trends data and Wikipedia topic view times [12, 13].

There are several stylized facts in financial markets, and a well-known one is the volatility clustering, i.e., the long-range time correlation of volatilities [2, 3, 14, 15]. Statistical properties of the price fluctuations and cross-correlations between individual stocks are topics of interest [16, 17], not only scientifically for unveiling the complex structure and internal interactions of the financial system, but also practically for the asset allocation and portfolio risk estimation [18, 19]. In the past years, much effort has been made to identify the business sectors from the cross-correlation matrix with the random matrix theory (RMT) [4, 7, 20–22]. The financial network has been gaining increasing interests, and it is helpful for understanding the interaction structures of the financial markets [23–25]. Recently, a new approach is proposed, which combines the cross-correlation decomposition based on the RMT theory with various methods in complex networks. It may not only identify the business sectors of the financial markets, but also characterize the interactions between the business sectors [26]. To further understand the financial dynamics, one may consider a higher-order time correlation, i.e., the return-volatility correlation [5, 6, 27, 28]. A negative return-volatility correlation, which is the so-called leverage effect, is observed in almost all stock markets in the world [29–32]. However, a positive return-volatility correlation is detected in Chinese stock markets, which is now called the anti-leverage effect [6, 27]. The leverage and anti-leverage effects are important for the risk management and optimal portfolio choice [5, 33].

To investigate the dynamic behavior of a time series in multi time scale, the Fourier spectral analysis and wavelet approach are two common methods. For the Fourier spectral analysis, there are some crucial restrictions, i.e., the dynamic system must be linear and the data should be periodic or stationary. Otherwise, the resulting spectrum will be physically not so meaningful [34]. For the wavelet analysis, a filter function should be selected beforehand, and one may only obtain a physically meaningful interpretation to linear phenomena [35]. As the time series of returns in financial markets are nonlinear and complex, the Fourier spectral analysis and wavelet approach may give misleading results [35].

Therefore, searching for a new technique to analyze the nonlinear and non-stationary time series is challenging. The empirical mode decomposition (EMD) appears to be a novel method in this respect [35, 36]. With the EMD method, a time series can be decomposed into a small number of intrinsic mode functions (IMFs), which are derived based on the local characteristic time scale of the data itself and describe the dynamic behavior from high frequency to low frequency [35–38]. All the IMFs are orthogonal to each other [35, 39]. The EMD method provides us the ability to analyze the dynamics of financial markets in intrinsic multi time scale [34]. An IMF is derived as a function having the same number of zero-crossings and extrema, and also having symmetric envelopes defined by the local maxima and minima, respectively [35, 40]. The cycle of each IMF can be regarded as an indicator of repeating patterns specific to recurrence events [39]. These events are meaningful for better understanding the raw data [41–43].

The EMD method was initially proposed to study the movement of the ocean waves [35, 38], and then successfully applied in different areas. One important application of this method is in social science, such as for the investigation of the dengue haemorrhagic fever [44], the crude oil price [38, 45] and the financial markets. In the past years, with the EMD method, the phase distribution and phase correlation of financial time series have been studied [39, 40], the damped oscillations in the ratios of stock market indices has been analyzed [39]. The financial crisis forecasting and foreign exchange rate forecasting have been investigated with this method, and the results are significantly improved compared with those obtained with conventional neural networks [41, 45]. The EMD method is very useful for both theoreticians and practitioners [46–48].

The spatial and temporal structures of financial markets in multi time scale are important, but so far have not been touched so much. Our motivation in this paper is to investigate the spatial and temporal structures of financial markets in multi time scale with the EMD method, and to understand their intrinsic dynamic mechanisms. After analyzing the probability distribution of returns, auto-correlation of volatilities and persistence probability of volatilities for different modes, we conclude that these basic characteristics are rather robust in multi time scale. However, the cross-correlation between individual stocks and the return-volatility correlation are time scale dependent. We uncover that the structure of business sectors in a stock market is mainly governed by the fast mode when returns are sampled at a couple of days, while by the medium mode when returns are sampled at dozens of days. More importantly, the leverage and anti-leverage effects are dominated by the medium mode.

## Materials

To investigate the cross-correlations between individual stocks in a stock market, the data set should include as many stocks as we may obtain. On the other hand, the absent data points for each stock should be as few as possible. With these considerations, we have collected the data of the daily closing prices of individual stocks for four stock markets. The price data of 174 stocks in the Shanghai Stock Exchange (SHSE) are from Jan., 1997 to Nov., 2007, with 2633 data points in total. The price data of 162 stocks in the Taiwan Stock Exchange (TWSE) are from Jan., 2003 to Apr., 2011, with 2000 data points in total. The price data of 158 stocks in the Hong Kong Stock Exchange (HKSE) are from Jan., 2003 to Apr., 2011, with 2000 data points in total. The price data of 246 stocks in the New York Stock Exchange (NYSE) are from Jan., 1990 to Dec., 2006, with 4286 data points in total.

For the stock market indices, the daily data of the German DAX are from 1959 to 2009 with 12407 data points. The daily data of the S&P 500 index are from Jan., 1990 to Dec., 2006 with 4286 data points. The daily data of the Hang Seng index (HSI) in Hong Kong are from 1990 to 2011 with 5472 data points. The daily data of the Taiwan Weighted index (TWII) are from 1997 to 2011 with 3571 data points. The daily data of the Shanghai Composite index (SHCI) are from 1990 to 2009 with 4482 data points, and the daily data of the Shenzhen Composite index (SZCI) in the Shenzhen Stock Exchange (SZSE) are from 1991 to 2009 with 4435 data points. All these data are obtained from Yahoo! Finance (finance.yahoo.com).

## Methods and Results

### Basic dynamic characteristics

In this section, we analyze the probability distribution of returns, auto-correlation of volatilities and persistence probability of volatilities. We denote the price of a stock index at time *t*′ as *P*(*t*′), then its logarithmic price return over a time interval Δ*t* is defined by

$$R\left(t^{\prime },\Delta t\right)\equiv ln\;P\left(t^{\prime }+\Delta t\right)-ln\;P\left(t^{\prime }\right).$$ (1)

**Fig 1:**
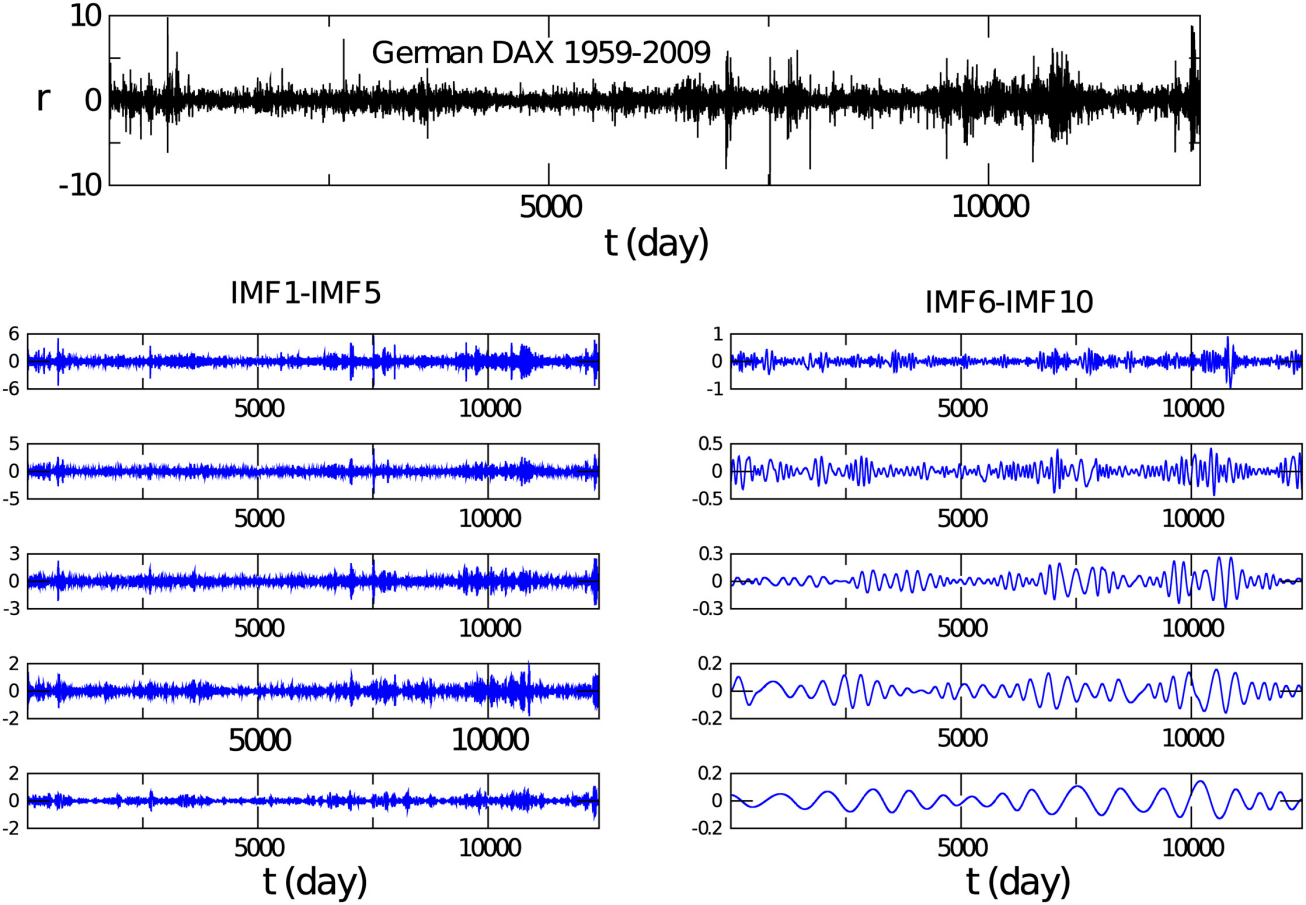
The time series of returns for the German DAX index, and the first ten IMFs decomposed with the EMD method. The left graph describes the 1st IMF to the 5th IMF from top to bottom, and the right one displays the 6th to 10th IMFs from top to bottom.

△*t* is first set to be one day, and the effect of different △*t* will be investigated in the last subsection. To ensure that the results are independent of the fluctuation scales of different financial indices, we introduce the normalized price return

$$r\left(t^{\prime }\right)=\left(R\left(t^{\prime }\right)-\langle R\rangle \right)/\sigma ,$$ (2)

where 〈⋯〉 represents the time average over time *t*′, and $\sigma =\sqrt{\langle R^{2}\rangle -{\langle R\rangle }^{2}}$ is the standard deviation of *R*(*t*′) [7]. With the EMD method [35, 36, 38], the time series of the price returns of each financial index is decomposed into a small number of intrinsic mode functions, i.e., the so-called IMFs, which are derived based on the local characteristic time scale of the data itself and characterize the price motion from high frequency to low frequency. But the IMFs are not exact periodic functions, and the cycle and amplitude of each IMF fluctuate within a certain range during the time evolution. To be clearer, we take the German DAX as an example. The time series of returns for this index is decomposed into thirteen IMFs from high frequency to low frequency. In Fig 1, the first ten IMFs are displayed. We observe that the average amplitude of an IMF monotonously decreases from high frequency to low frequency. The average cycle of each IMF is then computed [38]. For the first three IMFs, the cycles are 2.9, 5.5 and 9.4 days, respectively. For the fourth to the eighth IMFs, the cycles are respectively 16.5, 31.4, 59.9, 118.2 and 217.6 days. Roughly, the average cycle obeys a double increase from high frequency to low frequency.

The IMFs may then be grouped into three modes, i.e., the fast mode, medium mode and slow mode. The sum of the first three IMFs whose cycles are below two working weeks is identified as the fast mode. The sum of the fourth IMF to the eighth IMF is called the medium mode. Considering that the average cycle of the ninth IMF is larger than one working year, we identify the sum of the ninth to the last IMF as the slow mode.

**Fig 2:**
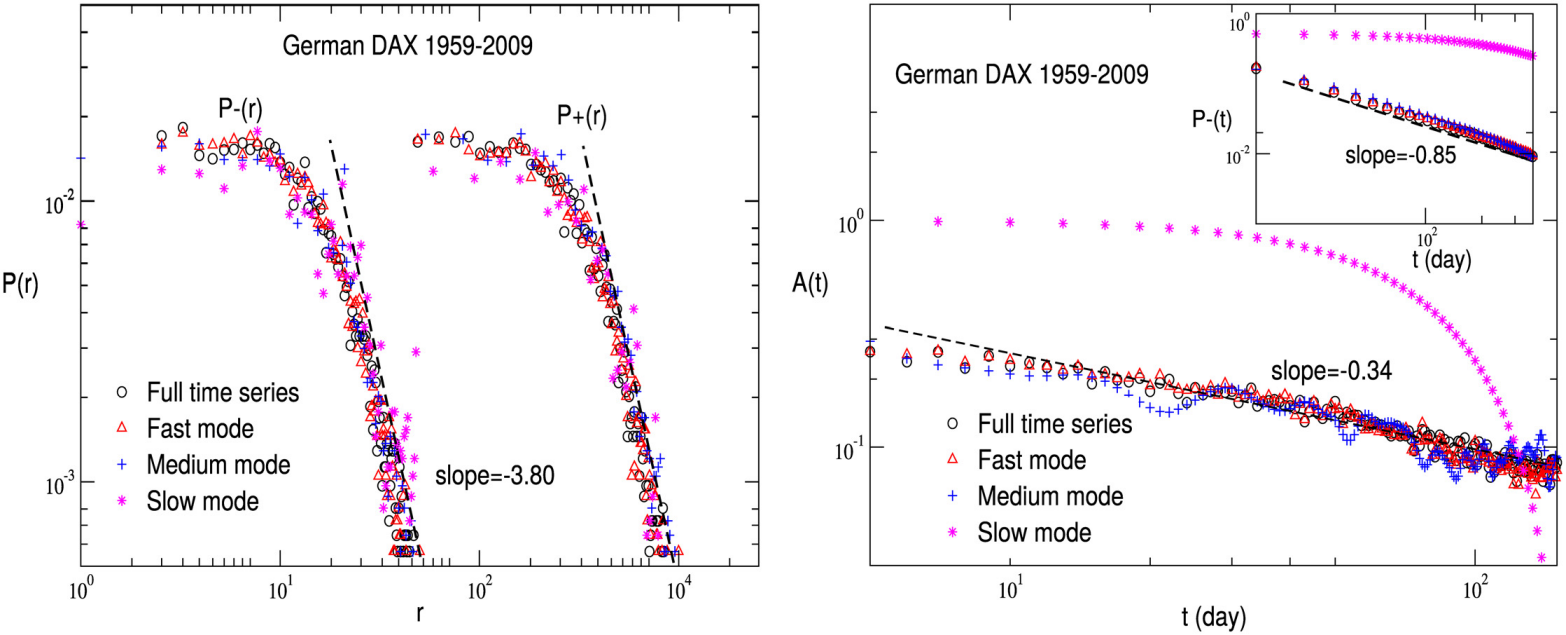
The basic characteristics of the German DAX index. (**a**) The probability distribution of returns for the full time series, fast mode, medium mode and slow mode is respectively displayed for the German DAX index. (**b**) The auto-correlation function and persistence probability of volatilities are shown.

The probability distributions of positive and negative returns are computed for the full time series, fast mode, medium mode and slow mode, respectively. The results for the German DAX are shown in Fig 2(a). All the curves exhibit a similar behavior with a fat-tail.

The auto-correlation function of volatilities is defined as

$$A\left(t\right)=[\langle |r\left(t^{\prime }\right)||r\left(t^{\prime }+t\right)|\rangle -\langle |r\left(t^{\prime }\right){|\rangle }^{2}]/A_{0},$$ (3)

with *A*_0_ = 〈∣*r*(*t*′)∣^2^〉−〈∣*r*(*t*′)∣〉^2^. It is well known that the volatility in financial dynamics is long-range correlated in time, i.e., *A*(*t*) decays by a power law [2, 3, 14, 15]. After analyzing the auto-correlation function of volatilities for all the IMFs, we observe that the curve for a single IMF shows a relatively large fluctuation. In Fig 2(b), therefore, *A*(*t*) is plotted for the full time series, fast mode, medium mode and slow mode of the German DAX. The curves of the full time series, fast mode and medium mode display a similar behavior. However, the behavior of the slow mode is deviating. In fact, as indicated in Fig 1, the average cycle of each IMF in the slow mode is larger than two hundred days. One may suffer from the long periodicity when computing the auto-correlation function for the slow mode.

We further explore the persistence probability of volatilities *P*_−_(*t*) which is defined as the probability that ∣*r*(*t*′+*t*)∣ has always been below ∣*r*(*t*′)∣ in time *t*. In general, *P*_−_(*t*) obeys a universal power law behavior, and describes the auto-correlation non-local in time, which may be independent of that local in time [27]. Considering that the slow mode has a long periodicity, we mainly focus on the behaviors of the fast mode and medium mode. As shown in the inset of Fig 2(b), the curves for the full time series, fast mode and medium mode follow a similar behavior.

Briefly speaking, the basic properties of a financial market, such as the probability distribution of returns, auto-correlation of volatilities and persistence probability of volatilities, are rather robust in multi time scale, at least for the fast mode and medium mode. Among these characteristics, the long-range auto-correlation of volatilities is dominating. It is reported that the cycle of each IMF can be regarded as an indicator of repeating patterns specific to recurrence events [39]. In other words, although these events occurs in different intrinsic time scales, their volatilities are similarly long-range correlated in time in the statistical sense [2, 3, 14, 15].

### Structure of business sectors

Following the notations in Eqs (1) and (2), the logarithmic price return of the *i*-th stock is denoted by *R*_*i*_(*t*′), and the normalized one by *r*_*i*_(*t*′). *r*_*i*_(*t*′) for △*t* = 1 is then decomposed into a small number of IMFs with the EMD method, and the typical number of IMFs is eleven to thirteen.

The elements of the equal-time cross-correlation matrix *C* are defined by

$$C_{ij}\equiv \langle r_{i}\left(t^{\prime }\right)r_{j}\left(t^{\prime }\right)\rangle ,$$ (4)

which measure the correlations between the price returns of individual stocks *i* and *j*. According to the definition, *C* is a real symmetric matrix with *C*_*ii*_ = 1, and *C*_*ij*_ is valued in the range [-1,1].

The Wishart matrix is derived from non-correlated time series. Assuming that there are *N* time series with a length *T*, statistical properties of such random matrices are well understood [49, 50]. In the limit *N* → ∞ and *T* → ∞ with *Q* ≡ *T*/*N* ⩾ 1, the probability distribution *P*_*rm*_(*λ*) of the eigenvalue *λ* is given by [49, 50]

$$P_{rm}\left(\lambda \right)=\frac{Q}{2\pi }\frac{\sqrt{\left(\lambda_{max}^{ran}-\lambda \right)\left(\lambda -\lambda_{min}^{ran}\right)}}{\lambda },$$ (5)

and the lower and upper bounds of *λ* are

$$\lambda_{min(max)}^{ran}={\left[1\pm \left(1/\sqrt{Q}\right)\right]}^{2}$$ (6)

For a real dynamic system, large eigenvalues deviating from *P*_*rm*_(*λ*) of the Wishart matrix imply that there exist non-random interactions. Both mature and emerging stock markets show such a phenomenon [4, 21, 51]. In our notations, the eigenvalues are arranged in the order of *λ*_*α*_ > *λ*_*α*+1_, with *α* = 0,…,*N*−1, with *N* being the number of stocks. The eigenmodes for the large eigenvalues are dominated by a community of stocks, usually associated with a business sector. Taking into account the signs of the components of the eigenvectors, the sector of *λ*_*α*_ may be further separated into two subsectors, i.e., the positive and negative subsectors [52, 53].

The cross-correlation between two stocks can be decomposed into different eigenmodes [26],

$$C_{ij}=\sum_{\alpha =1}^{N}\lambda_{\alpha }C_{ij}^{\alpha },\;C_{ij}^{\alpha }=u_{i}^{\alpha }u_{j}^{\alpha },$$ (7)

where $u_{i}^{\alpha }$ is the *i*-th component in the eigenvector of *λ*_*α*_, and $C_{ij}^{\alpha }$ represents the cross-correlation in the *α*-th eigenmode. In order to uncover the interaction structure in different eigenmodes, we introduce the mode cross-correlation matrix

$$C_{mode,ij}=\sum_{\alpha }^{mode}\lambda_{\alpha }C_{ij}^{\alpha }.$$ (8)

For the market mode, $C_{mar,ij}=\lambda_{0}C_{ij}^{\alpha }$. For the sector mode, $C_{sec,ij}=\sum_{\alpha =1}^{n-1}\lambda_{\alpha }C_{ij}^{\alpha }$, and *n* is the number of large eigenvalues *λ*_*α*_, i.e., $\lambda_{\alpha }>\lambda_{max}^{ran}$. Usually, *n* is less than twenty. For the random mode, we typically take $C_{ran,ij}=\sum_{\alpha =50}^{N-1}\lambda_{\alpha }C_{ij}^{\alpha }$.

According to the cross-correlation decomposition, the global price movement of the entire market, the local price motion of business sectors and the background of the noisy correlations between individual stocks are described by the market mode, sector mode and random mode respectively. In fact, previous research reveals that one can not obtain really meaningful business sectors and their interactions from the market mode and random mode [26]. The nontrivial local interactions of the business sectors are mainly contained in the sector mode. Additionally, a business sector in the sector mode may split into two subsectors, which are anti-correlated each other. These two subsectors are called a cluster pair [26, 52, 53].

**Fig 3:**
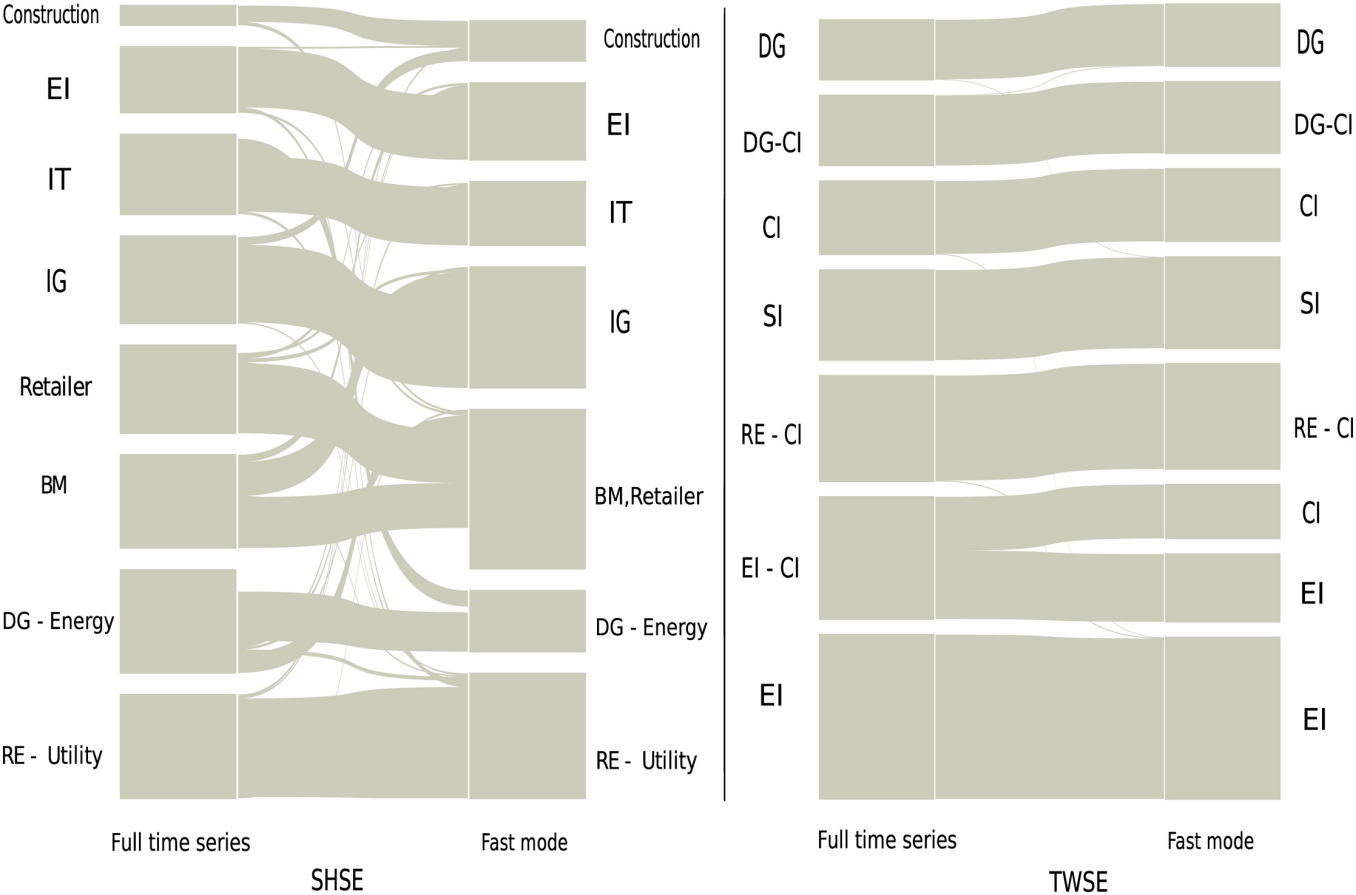
The business sectors of the full time series for the SHSE and TWSE markets are compared with those of the fast mode. DG-Energy and RE-Utility are cluster pairs for the SHSE market, while RE-CI, EI-CI and DG-CI are those for the TWSE market. The abbreviations are as follows. RE: Real estate; CI: Chemical industry; SI: Steel industry; IG: Industrial goods; EI: Electronic industry; IT: Technology; BM: Basic materials; Serv: Service; DG: Daily consumer goods.

Enlightened by the work in ref. [26], a methodology which combines the RMT theory with the planar maximally filtered graph and the alluvial diagram is introduced [54, 55]. The alluvial diagram is well applied to visualize the structural changes in network structures [55]. With this approach, we investigate the interaction structures of business sectors with the sector mode cross-correlation matrix, for the full time series, fast mode, medium mode and slow mode respectively. The alluvial diagrams of the business sectors for the SHSE and TWSE markets are shown in Fig 3. The height of a module is proportional to the number of its stocks. The module usually corresponds to a business sector, in which most of the stocks are running a same business. As shown in Fig 3, the structure of business sectors in the SHSE market for the fast mode is almost identical with that for the full time series. The identified cluster pairs are DG-Energy and RE-Utility. For the TWSE market, the behavior is similar.

**Fig 4:**
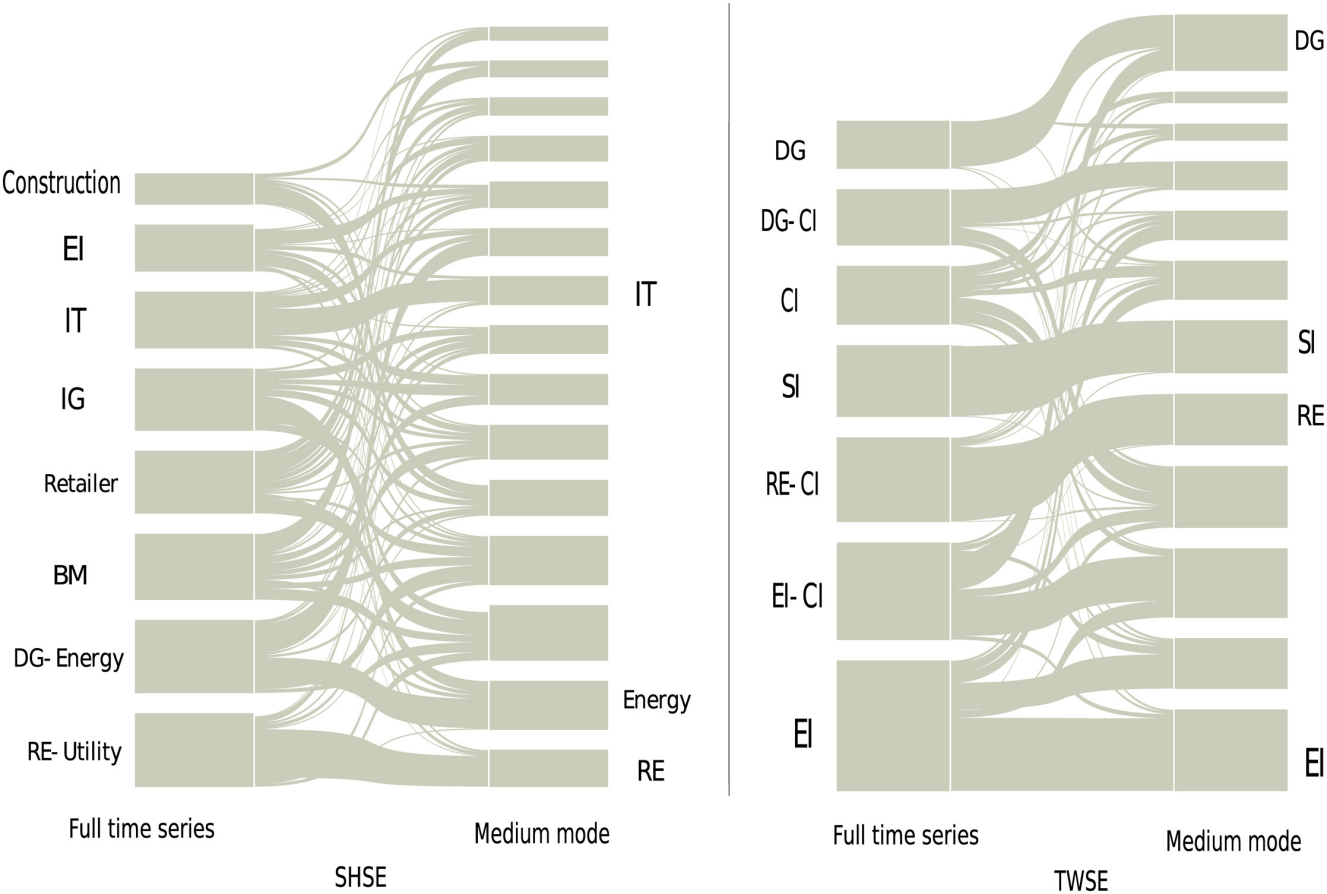
The business sectors of the full time series for the SHSE and TWSE markets are compared with the communities of the medium mode. Most of the communities of the medium mode could not be associated with the business sectors. The abbreviations are the same as in Fig 3.

For the medium mode, the identified communities are very different from those of the full time series. In addition, these communities can hardly be associated to business sectors. The alluvial diagrams are shown in Fig 4. Energy, Technology and Real estate for the SHSE, and Electronic industry, Real estate, Steel industry and Daily consumer goods for the TWSE are only exceptional, and these business sectors are the representative and important ones in their own stock market [26, 53]. For the slow mode, the communities are also different, and one could not identify any business sectors.

We have also examined the NYSE and HKSE markets, and our conclusion is that the structure of business sectors in a stock market is mainly governed by the fast mode. More precisely, according to the average cycle of each IMF computed in Subsec. Basic dynamic characteristics of Sec. Results, the price fluctuations whose time scales are below ten days, i.e., two working weeks, contribute the most to the structure of business sectors. This result is somewhat unexpected. A naive conjecture might be that the medium mode and slow mode should also contribute to the structure of business sectors, if not dominating, since the structure of business sectors is considered to be relatively stable during the time evolution.

**Fig 5:**
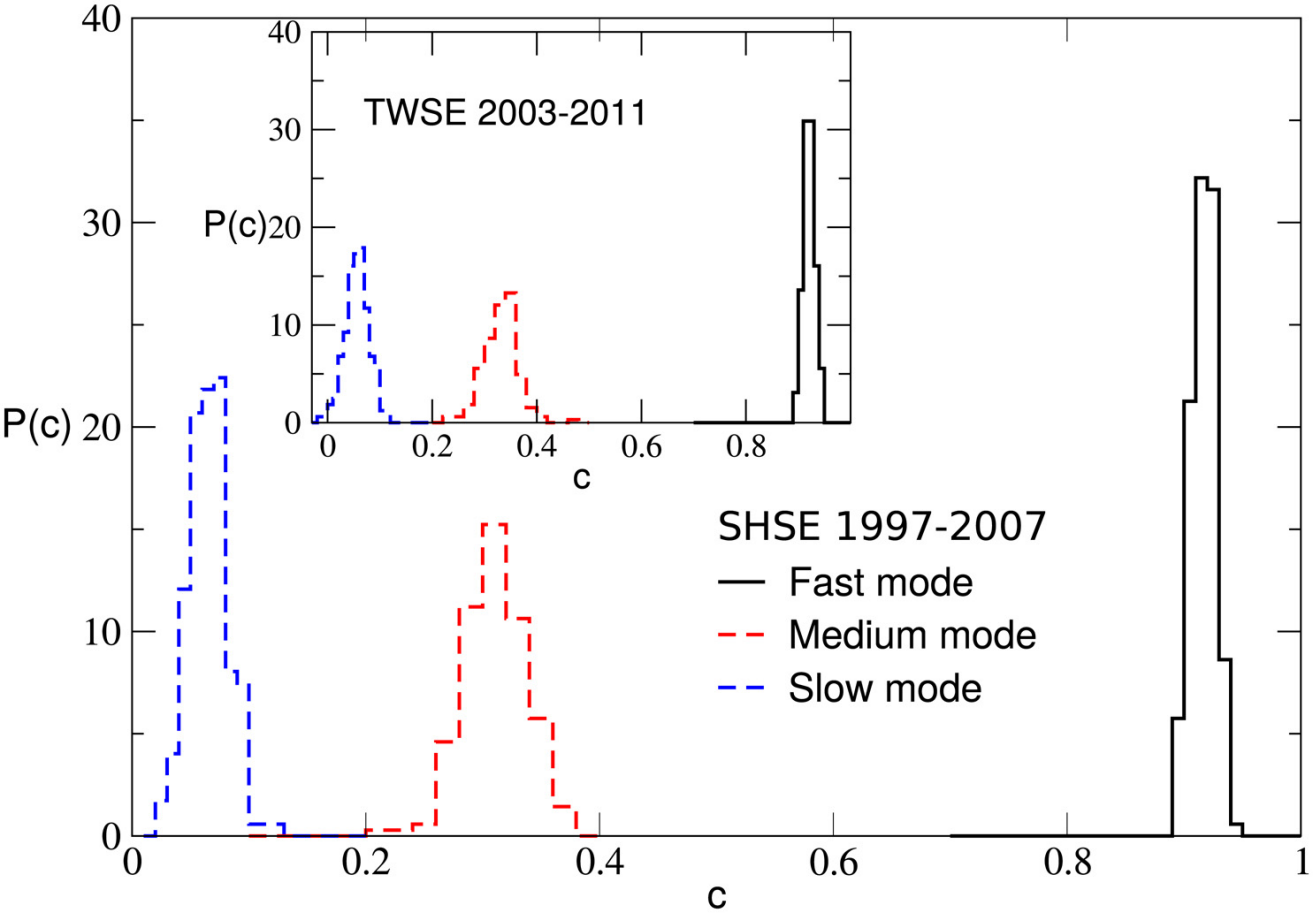
The probability distribution of the correlation *c* defined in Eq (9) for the fast mode, medium mode and slow mode. There are 174 stocks for the SHSE market, and 162 stocks for the TWSE market.

To obtain a better understanding of the above results, we compute the equal-time correlation between the full time series of returns and the fast, medium or slow mode, and denote it by

$$c=\langle r_{i}\left(t^{\prime }\right)r_{i}^{m}\left(t^{\prime }\right)\rangle ,$$ (9)

with $r_{i}^{m}\left(t^{\prime }\right)$ being the fast, medium or slow mode of *r*_*i*_(*t*′). The value of *c* may change for different stocks. In Fig 5, the probability distribution of the correlation *c* is shown for the SHSE and TWSE markets. The correlations for the fast mode are larger than those for the medium mode and much larger than those for the slow mode. The average value of *c* for the fast mode is 0.92 and 0.93 for the SHSE and TWSE respectively, while that for the medium mode is 0.30 and 0.32. The average value of *c* for the slow mode is about 0.05 for both the SHSE and TWSE markets. Therefore, the fast mode of *r*_*i*_(*t*′) looks almost identical to *r*_*i*_(*t*′), but the medium mode and slow mode are not. The results for the NYSE and HKSE markets are similar. This should be an important reason that for Δ*t* = 1, the structure of business sectors in a stock market is mainly governed by the fast mode.

### Return-volatility correlation

The return-volatility correlation function is usually defined by

$$L\left(t\right)=[\langle r\left(t^{\prime }\right)|r\left(t^{\prime }+t\right){|}^{2}\rangle -L_{0}]/Z,$$ (10)

with *Z* = 〈∣*r*(*t*′)∣^2^〉^2^ and *L*_0_ = 〈*r*(*t*′)〉〈∣*r*(*t*′)∣^2^〉. For *t* > 0, *L*(*t*) describes how the past returns affect the future volatilities. It was first discovered by Black that the past negative returns increase future volatilities, i.e., the return-volatility correlation is negative [29, 30], and this phenomenon is called the leverage effect. The leverage effect is observed in almost all the stock markets in the world. However, a positive return-volatility correlation, which is the so-called anti-leverage effect, is detected in the Chinese stock markets in the past years [6, 7, 27]. In very recent years, many researches have been devoted to the return-volatility correlation. Various macroscopical models and a recent microscopic model have been proposed [5, 7, 56–59]. In this paper, the EMD method provides a possible technique to analyze the return-volatility correlation in multi time scale.

**Fig 6:**
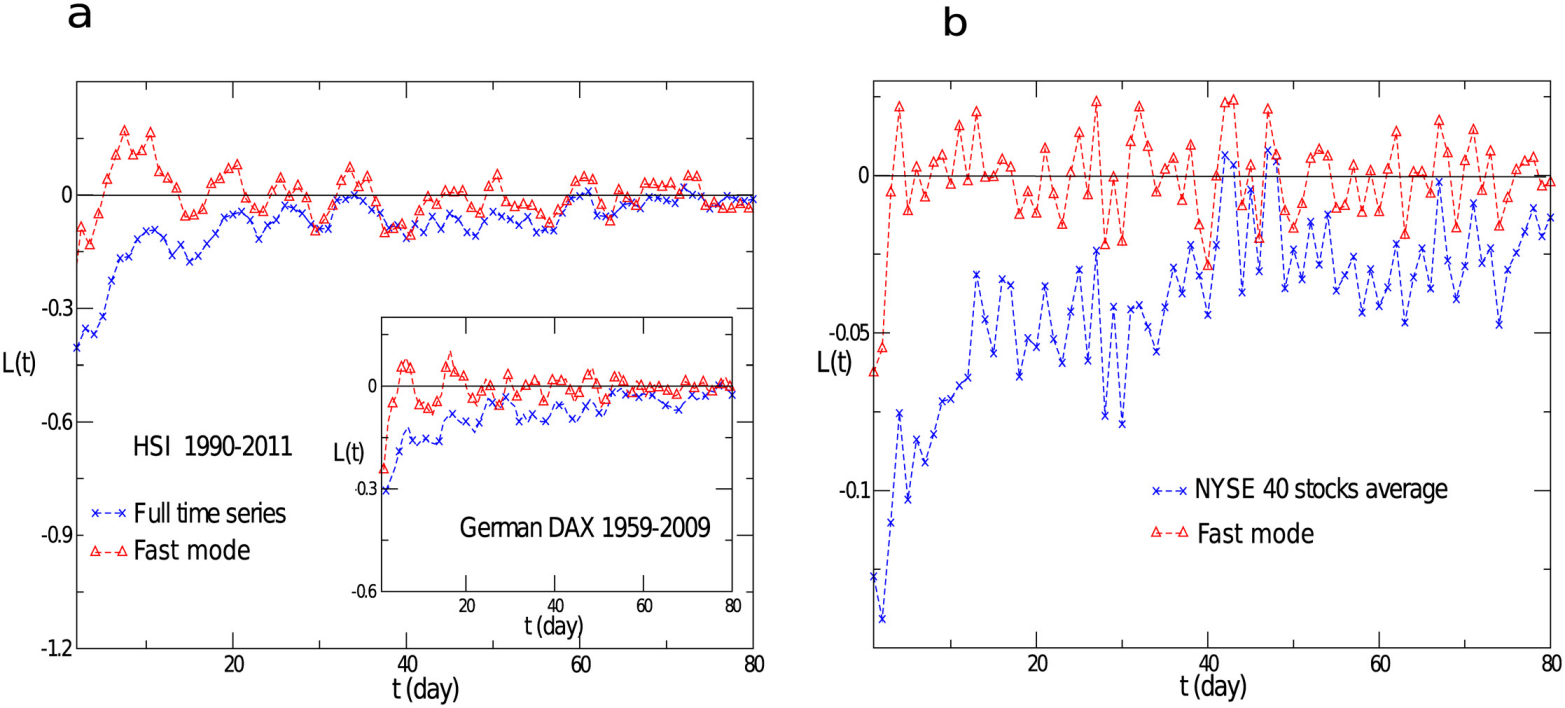
The return-volatility correlation function for the full time series and the fast mode. (**a**) The return-volatility correlation function for the HSI index in Hong Kong and the German DAX index, and that for the fast mode of each index. (**b**) The return-volatility correlation function averaged over the chosen 40 stocks for the NYSE market, and that of the fast mode.

Firstly, we calculate the return-volatility correlation function for the German DAX index and the HSI index in Hong Kong, respectively. As shown in Fig 6(a), the return-volatility correlation function of the full time series for the two indices exhibit a leverage effect, while that of the fast mode just fluctuates around zero. This is a surprising result, since the equal-time correlation between the full time series and fast mode is very large, over 0.92 in general. For the Chinese indices, i.e., the average of the SHCI and SZCI indices, it is observed that before the year 2000, it exhibited a strong anti-leverage effect, while after 2000, it gradually changed to the leverage effect [53]. For comparison, we concentrate our attention on the period from 1990 to 2000. Again, the return-volatility correlation function for the fast mode fluctuates around zero. We have also analyzed the return-volatility correlation for individual stocks. We choose 40 stocks from the NYSE market, which show a strong leverage effect, and the results are displayed in Fig 6(b). The return-volatility correlation of the fast mode also fluctuates around zero. Thus we conclude that the fast mode dose not contribute to the leverage or anti-leverage effect.

**Fig 7:**
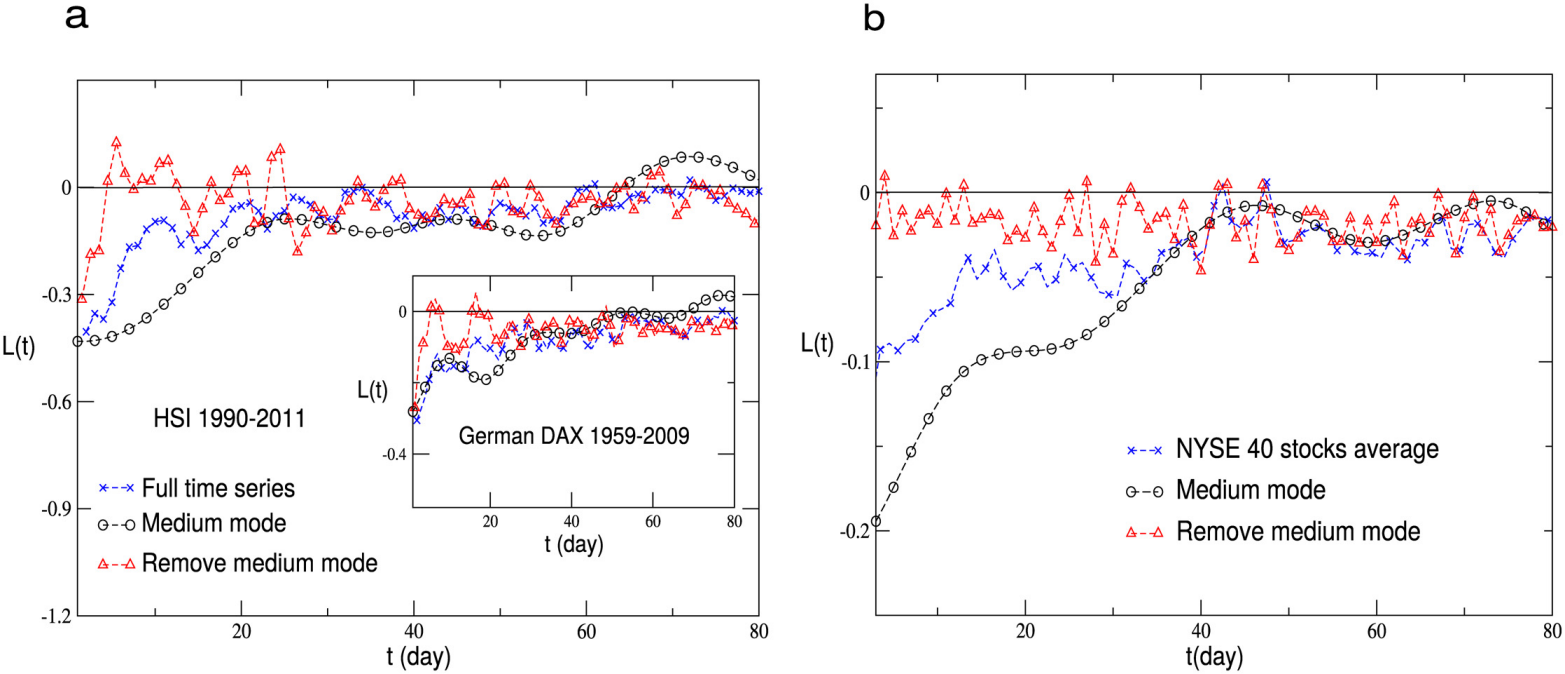
The return-volatility correlation function of the full time series, the medium mode and that after removing the medium mode. (**a**) The return-volatility correlation function of the full time series, the medium mode and that after removing the medium mode for the HSI index in Hong kong and the German DAX index. (**b**) The return-volatility correlation function of the full time series, the medium mode and that after removing the medium mode for the NYSE market. The results are averaged over the chosen 40 stocks.

After careful analyzing, we find that the medium mode contributes the most to the leverage and anti-leverage effects while the slow mode also does not. The average cycles are from fifteen days to over two hundred days. In Fig 7(a), the return-volatility correlation function is shown for the German DAX index and the HSI index in Hong Kong, and in Fig 7(b), the one averaged over the chosen 40 stocks for the NYSE market is displayed. In all these cases, the medium mode dominates the leverage effect. After removing the medium mode, the leverage effect is not detected. As shown in Fig 1, each frequency mode exhibits a quasi periodic behavior. Such a periodicity will be reflected in the return-volatility correlation function, especially for the medium and slow modes. Therefore, the curve for the medium mode in the figures is the average of the upper and lower envelopes of the original one.

Why the medium mode is important for the leverage and anti-leverage effects? Previous analysis reveals that large volatilities are dominating [27]. For example, the leverage effect for the DAX is mainly contributed from the volatilities ∣*r*(*t*′)∣ > 2*σ*, while the anti-leverage effect for the Chinese indices is essentially dominated by the volatilities ∣*r*(*t*′)∣ > 8*σ*. On the other hand, it is reported that the high frequency IMFs reflect the short-term fluctuations, while each sharp up or down of the medium frequency IMFs corresponds to a significant event [38]. These events may cause large volatilities.

Enlightened by these works, we generate six new time series of returns by removing the large volatilities ∣*r*(*t*′)∣ > 8*σ* for the SHCI and SZCI indices, and ∣*r*(*t*′)∣ > 2*σ* for the S&P 500, German DAX, HSI and TWII indices. All the new time series do not show a leverage or an anti-leverage effect. Then we compute the equal-time correlation between an IMF of a full time series and that of the new time series. In Table 1, the results are shown for the six stock market indices. We observe that the correlation is obviously smaller for the IMFs with medium frequencies. The ninth IMF is an exception. This may be due to its small amplitude, and even a minor contribution from the volatilities may bring a large fluctuation to the equal-time correlation. In other words, the large volatilities are mainly contained in the medium mode rather than in the fast or slow mode. Therefore, the fast mode and slow mode do not contribute to the leverage or anti-leverage effect. Both the effects are dominated by the medium mode, or mainly by the large volatilities whose time scales are above two working weeks.

**Table 1:** The equal-time correlation between an IMF of the full time series and that of the new time series. The new time series are generated by removing the large volatilities, i.e., ∣*r*∣ > 8*σ* for the SHCI and SZCI indices, and ∣*r*∣ > 2*σ* for the S&P 500, DAX, HSI and TWII indices.

	1	2	3	4	5	6	7	8	9	10
S&P 500	0.71	0.62	0.51	**0.43**	**0.39**	**0.35**	**0.31**	**0.09**	0.15	0.54
German DAX	0.68	0.57	0.51	**0.45**	**0.42**	**0.41**	**0.34**	**0.20**	0.12	0.54
HSI	0.67	0.56	0.54	**0.46**	**0.36**	**0.43**	**0.11**	**0.18**	0.22	0.41
TWII	0.71	0.56	0.52	**0.42**	**0.44**	**0.39**	**0.19**	**0.42**	0.68	0.70
SHCI	0.80	0.61	0.51	**0.67**	**0.73**	**0.57**	**0.44**	**0.43**	0.14	0.91
SZCI	0.93	0.82	0.78	**0.70**	**0.69**	**0.51**	**0.65**	**0.50**	0.74	0.67

### The effect of △*t*

**Fig 8:**
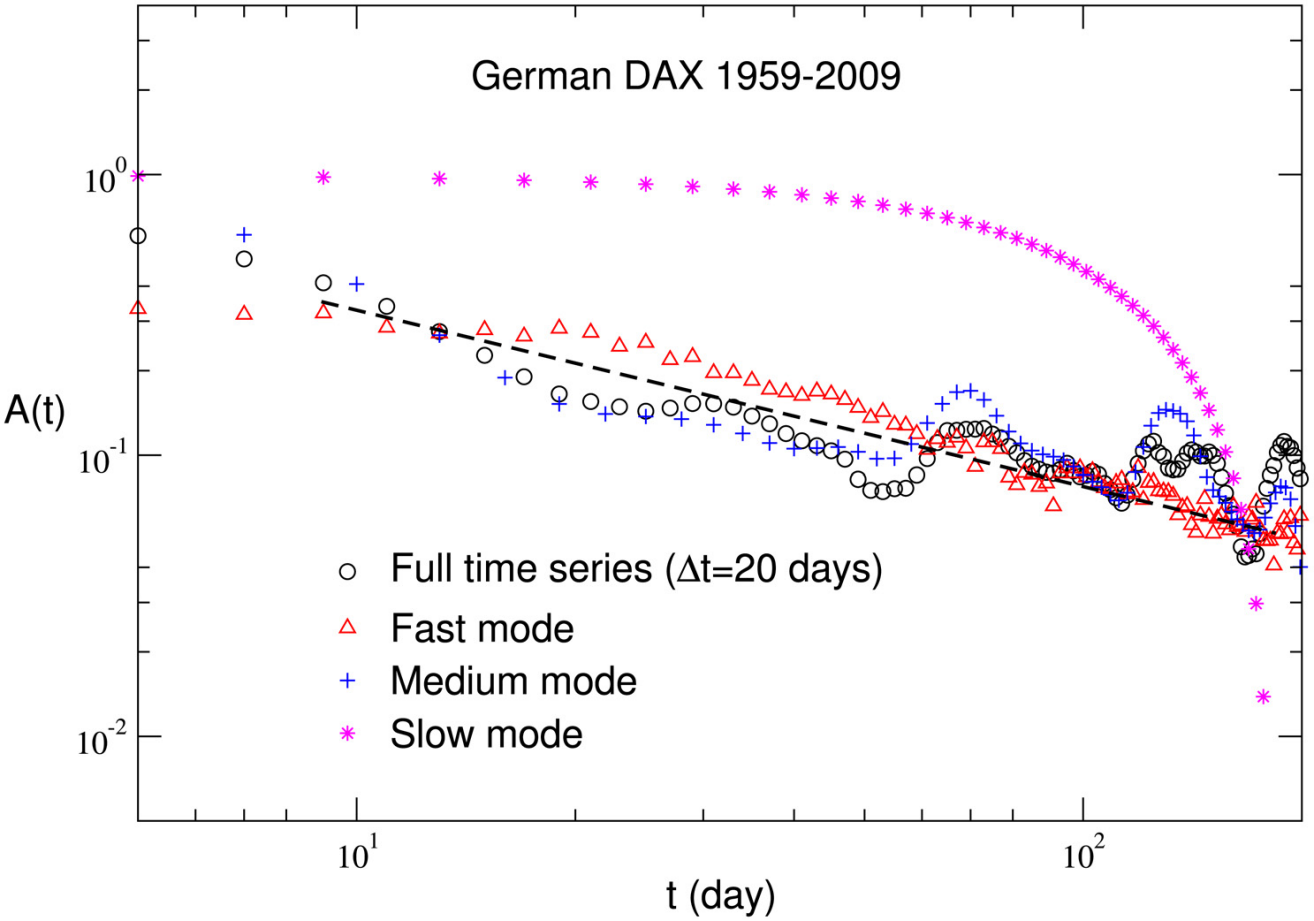
The auto-correlation function of volatilities for the full time series, fast mode, medium mode and slow mode of the German DAX. The value of △*t* is set to be 20 days.

To further investigate how the dynamic properties of different modes depend on the time scale, we change the value of △*t* in computing the price returns in Eq (1), for example, △*t* = 5, 10, 20 and 60 days. After analyzing the probability distribution of returns, the auto-correlation function and the persistence probability of volatilities for different △*t*, we find that the results of these three characteristics are consistent with those obtained for △*t* = 1. In other words, these characteristics are robust in multi time scale for different △*t*. In Fig 8, for example, the auto-correlation function of volatilities for the German DAX is shown for △*t* = 20. As discussed before, the deviation of the slow mode is possibly due to its long periodicity.

**Fig 9:**
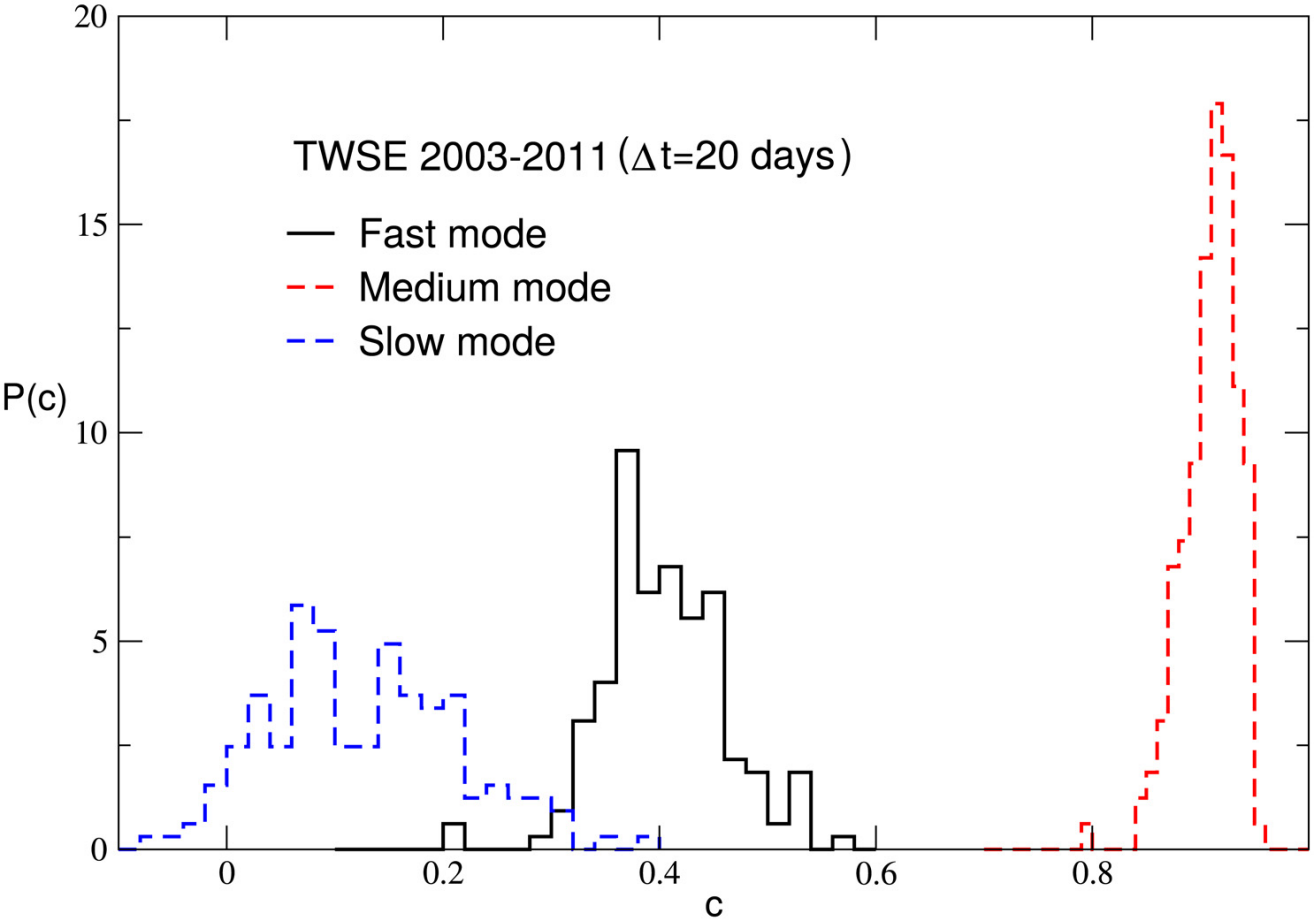
The probability distribution of the correlation *c* defined in Eq (9) for the fast mode, medium mode and slow mode. There are 162 stocks for the TWSE market, and △*t* = 20 days.

Following the procedure in Subsec. Structure of business sectors of Sec. Results, for a particular △*t*, the normalized logarithmic price return of each stock is decomposed into a small number of IMFs. The equal-time correlation between the full time series of returns and the fast, medium or slow mode are computed. The probability distributions of the correlation *c* in Eq (9) for △*t* = 20 days are displayed for the TWSE market in Fig 9, and the results are different from those for △*t* = 1. For △*t* = 20, the mode who has the largest equal-time correlation with the full time series is not the fast mode but the medium mode. The average value of *c* for the fast mode and the slow mode is 0.39 and 0.12 respectively, while that for the medium mode is 0.90.

**Fig 10:**
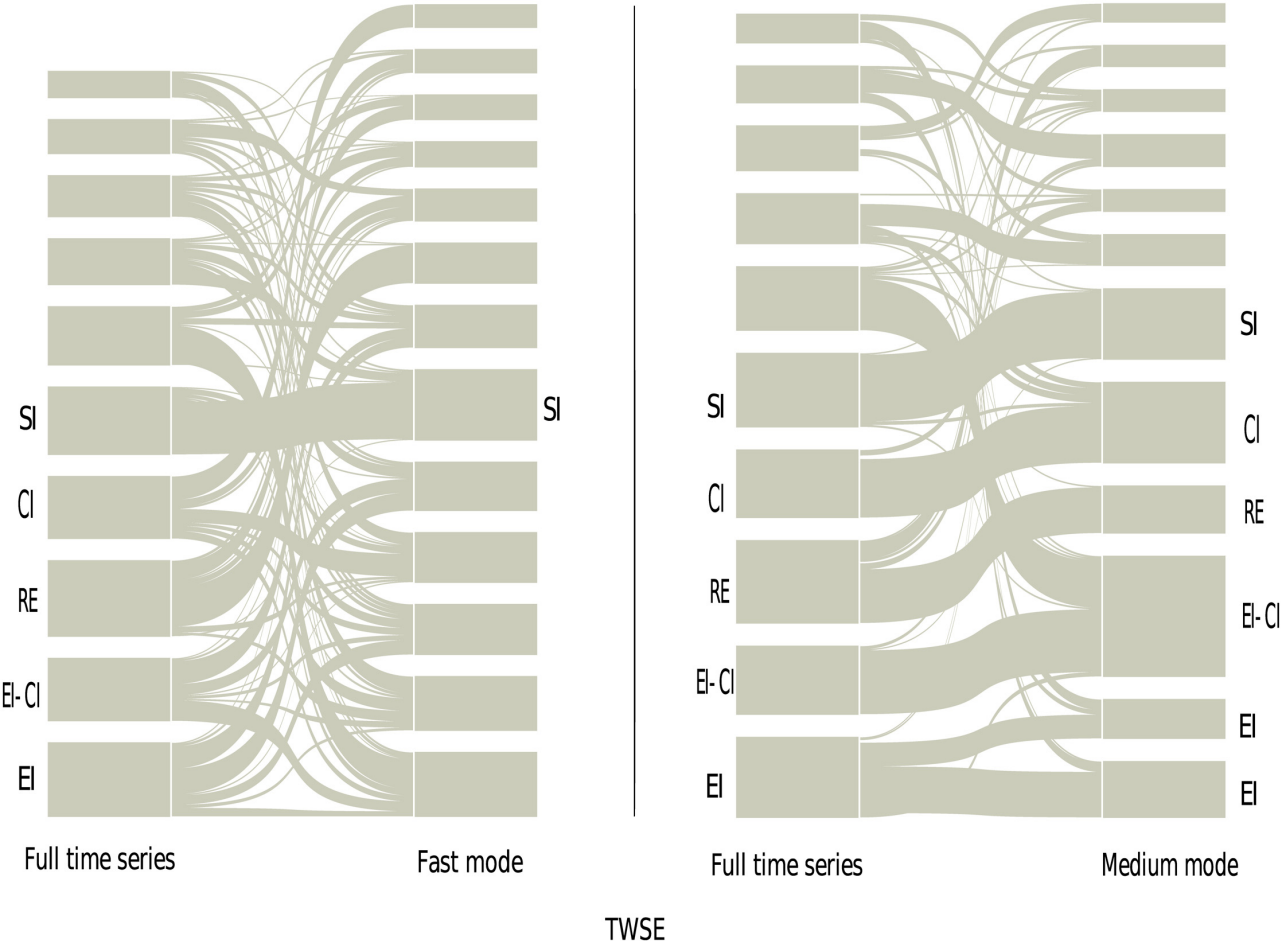
The business sectors of the full time series for the TWSE market are compared with those of the fast mode and medium mode for △*t* = 20 days. The abbreviations are the same as in Fig 3. The module that does not have a name is the one we could not identify.

To obtain the interaction structure of communities, again, the methodology which combines the RMT theory with the planar maximally filtered graph and the alluvial diagram is applied. With this approach, the alluvial diagrams of the business sectors for the four stock markets have been analyzed. In Fig 10, the results for the TWSE market are displayed. All the business sectors for the full time series can be discovered in the medium mode. For the fast mode and slow mode, the identified communities are very different from those of the full time series, and can hardly be associated to business sectors. The other three stock markets also show a similar phenomenon.

We observe that for a particular △*t*, the IMF with the largest average amplitude has the largest equal-time correlation with the full time series. We call this IMF the most correlated one, and it changes with △*t*. Further, the period of volatility for the most correlated IMF is approximately equal to △*t*. Here we should note that the period of volatility is equal to half of the period of return. For △*t* = 20 days, for example, the period of volatility for the fourth IMF is about twenty days. The practical computation shows that the fourth IMF is indeed the most correlated one. This should be an explanation that for △*t* = 20 days, the business structure is mainly governed by the medium mode.

**Fig 11:**
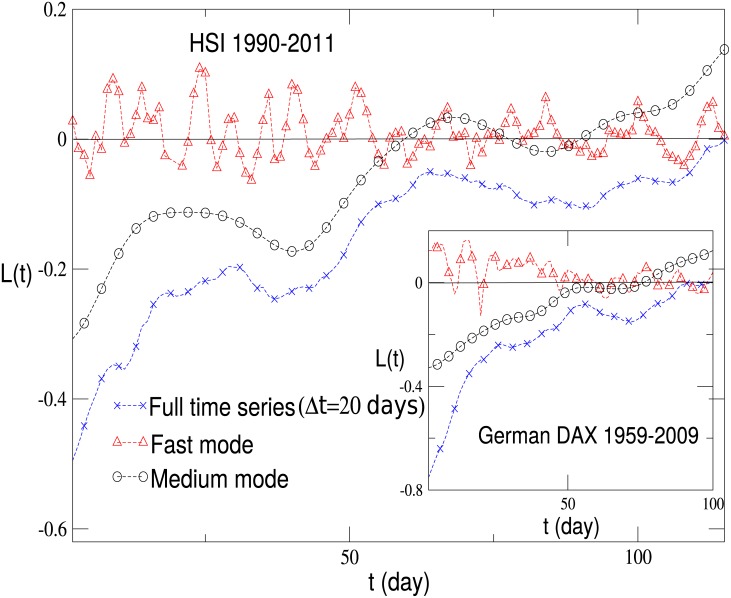
The return-volatility correlation function of the full time series, fast mode and medium mode for the HSI index in Hong kong and the German DAX index with △*t* = 20 days.

Finally, the return-volatility correlation functions for different △*t* are computed, and the results for the HSI index and the German DAX index with △*t* = 20 are shown in Fig 11. It remains that the medium mode contributes the most to the leverage effect, while the return-volatility correlation function of the fast mode fluctuates around zero.

## Discussion

In this paper, we explore the intrinsic multi-scale dynamic behavior of complex financial systems, based on the daily data of individual stock prices and financial indices in five typical stock markets, i.e., the NYSE, SHSE, SZSE, HKSE and TWSE markets. With the EMD method, the time series of the price returns of each stock or index can be decomposed into a small number of intrinsic mode functions, i.e., the so-called IMFs. The decomposition is based on the local characteristic time scale of data itself, and the IMFs represent the price motion from high frequency to low frequency. After analyzing the probability distribution of returns, auto-correlation of volatilities and persistence probability of volatilities for different modes, we conclude that these basic characteristics are rather robust in multi time scale. However, the cross-correlation between individual stocks and the return-volatility correlation are time scale dependent. The structure of business sectors in a stock market is mainly governed by the fast mode when returns are sampled at a couple of days, while by the medium mode when returns are sampled at dozens of days. More importantly, the leverage and anti-leverage effects are dominated by the medium mode.
